# Purification, identification and characterization of Nag2 *N-*acetylglucosaminidase from *Trichoderma virens* strain mango

**DOI:** 10.1186/s40529-022-00344-x

**Published:** 2022-05-17

**Authors:** Jheng-Hua Huang, Feng-Jin Zeng, Jhe-Fu Guo, Jian-Yuan Huang, Hua-Chian Lin, Chaur-Tsuen Lo, Wing-Ming Chou

**Affiliations:** Department of Biotechnology, National Formosa University, Yunlin, 632 Taiwan, ROC

**Keywords:** *Trichoderma virens*, Chitinase, *N*-acetylglucosaminidase, Exochitinase, *N*-acetylglucosamine

## Abstract

**Background:**

*N*-acetylglucosaminidase (NAGase) could liberate *N*-acetylglucosamine (GlcNAc) from GlcNAc-containing oligosaccharides. *Trichoderma* spp. is an important source of chitinase, particularly NAGase for industrial use. *nag1* and *nag2* genes encoding NAGase*,* are found in the genome in *Trichoderma* spp. The deduced Nag1 and Nag2 shares ~ 55% homology in *Trichoderma virens.* Most studies were focus on Nag1 and *nag1* previously.

**Results:**

The native NAGase (TvmNAG2) was purified to homogeneity with molecular mass of ~ 68 kDa on SDS-PAGE analysis, and identified as Nag2 by MALDI/MS analysis from an isolate *T. virens* strain mango. RT-PCR analyses revealed that only *nag2* gene was expressed in liquid culture of *T. virens*, while both of *nag1* and *nag2* were expressed in *T. virens* cultured on the plates. TvmNAG2 was thermally stable up to 60 °C for 2 h, and the optimal pH and temperature were 5.0 and 60–65 °C, respectively, using *p*-nitrophenyl-*N*-acetyl-*β*-D-glucosaminide (*p*NP-NAG) as substrate. The hydrolytic product of colloidal chitin by TvmNAG2 was suggested to be GlcNAc based on TLC analyses. Moreover, TvmNAG2 possesses antifungal activity, inhibiting the mycelium growth of *Sclerotium rolfsii*. And it was resistant to the proteolysis by papain and trypsin.

**Conclusions:**

The native Nag2, TvmNAG2 was purified and identified from *T. virens* strain mango, as well as enzymatic properties. To our knowledge, it is the first report with the properties of native *Trichoderma* Nag2.

## Background

Chitin, a homopolymer of 1,4-ß-linked *N*-acetylglucosamine (GlcNAc), is ranked as the second natural carbon source and nitrogenous organic compound after cellulose and protein, respectively. It is produced by living organisms, such as arthropods, mollusks, fungi and algae, on the order of 10^10^–10^14^ tons annually (Dhillon et al. 2013; El Knidri et al. 2018; Hamed et al. 2016; Ibitoye et al. 2018; Kaur and Dhillon 2015). Abundant chitinous waste may cause environmental issue; nevertheless, chitinolytic enzymes are capable to converse the renewable chitinous waste to the functional chitooligosaccharides or GlcNAc. They are further applied in food, cosmetic and dermatological, pharmaceuticals and biomedical etc. fields (Aam et al. 2010; Casadidio et al. 2019; Chen et al. 2010; Hamed et al. 2016).

The reported chitinolytic enzymes include endochitinases and exochitinases. Endochitinases, the member of glycoside hydrolase (GH) family 18 or 19, randomly split internal β-1,4-glycosidic bonds of chitin to release *N*-acetyl chitooligosaccharides. And exochitinases are further subclassfied into chitobiosidases (EC 3.2.1.29) and *N*-acetyl β-1,4-D-glucosaminidases (also termed *N*-acetylglucosaminidase, NAGase) (EC 3.2.1.30). Chitobiosidases release diacetylchitobiose units from the nonreducing terminal end of chitin or *N*-acetyl chitooligosaccharides stepwise. NAGase could liberate GlcNAc from nonreducing terminal residues of chitins, *N*-acetyl chitooligosaccharides and diacetylchitobiose.

GlcNAc are commonly applied to treat Osteoarthritis, as well as glucosamine (GlcN), its deacetylated derivative (Crolle and D'este 1980). They are also widely used in food, and cosmetics industries (Chen et al. 2010; Liu et al. 2013), and potential used for the production of ethanol (Inokuma et al. 2013). The industrial GlcN supply is mainly from hydrolysis of chitin by chemical method with HCl, and GlcNAc is formed after acetylation of GlcN with acetic anhydride. However, those process is not friendly to environment. The hydrolysis of chitin by chitinolytic enzymes from microorganism to produce GlcNAc is expected to be an alternative and ongoing way (Liu et al. 2013).

*Trichoderma* spp. well recognized as biocontrol antagonizes pathogenic fungi by composite mechanisms, including secretion of cell wall degrading enzymes, chitinolytic enzymes and β-1,3-glucanases (Sood et al. 2020). *Trichoderma* spp. is one of important sources to produce chitinolytic enzymes, particularly NAGase. Based on the protein structure and catalytic mechanism, NAGase from various sources are classified into GH3, GH20 and GH84 of family in CAZy database.

The abundant putative genes (20–36 genes) encoding endochitinase of GH18 are in the genome of *T. virens*, *T. atroviride* or *T. reesei*, compared to other fungi (Kubicek et al. 2011). And two *nag1* and *nag2* genes coding for NAGase of GH20*,* are found in the genome of above *Trichoderma* spp. The deduced protein sequence of *T. virens nag1* shares ~ 55% to *T. virens nag2.* The deduced protein sequence of *nag1* from *T. virens*, *T. atroviride* and *T. reesei* shared > 80% identity to each other, as well as *nag2*, > 80% identity. The physiological role of NAGase in *Trichoderma* spp. is not so clear. It was revealed that NAGase (either Nag1 or Nag2) are necessary for the growth of *T. atroviride* on chitin or chitobiose by using the knock-out study with Δ*nag1* and Δ*nag2* (López‐Mondéjar et al. 2009). The chitinolytic enzymes, endochitinase and NAGase from *Trichoderma* spp. have been characterized since last 2–3 decade. However, they were mostly done before the protein identification by LC/MS/MS or MADI/MS available. The enzymatic property of Nag1 in recombinant or native form was reported (Chen et al. 2015), while little was known with Nag2.

The ability to hydrolyse chitin by different *Trichoderma* spp. is relatively diverse. Over two hundred of *Trichoderma* isolates were surveyed in this study using the chitin-containing plate assay. The selected *T. virens* strain mango exhibited the highest chitinase activity. The induction days of *Trichderma* chitinases including endochitinase and NAGase were assessed. NAGase from *T. virens* strain mango (TvmNAG2) was subsequently purified, and identified as Nag2. The purified native TvmNAG2 was characterized and its potential application was thereby discussed. To our knowledge, it is the first report with enzymatic properties of native *Trichoderma* Nag2. And the production of GlcNAc by TvmNAG2 was preliminarily evaluated. Moreover, a *nag2* gene coding for Nag2 (TvmNAG2) was obtained by PCR-cloning.

## Methods

### Trichoderma *strains and chemicals*

*Trichoderma* isolates used in this study was obtained from Prof. Lo’s lab in Department of Biotechnology at National Formosa University, Taiwan. The isolates were maintained and sporulated on potato dextrose agar plates at 28 °C for 7 days. Chitin from the crab shells, chitosan (DA 85%), carboxymethylcellulose (CMC), starch, 3,5-dinitrosalicylic acid (DNS), *p*-nitrophenyl, *p*-nitrophenyl-*N*-acetyl-*β*-D-glucosaminide (*p*NP-NAG), GlcNAc, and 4-MU-α-GlcNAc3 were purchased from Sigma Chemicals Co. (St. Louis, MO, USA). *N*, *N*’-diacetylchitibiose was from Toronto Research Chemicals (Toronto, ON. Canada).

### Preparation of colloidal chitin and glycol chitin

20 gof powder crab chitin was mixed with 100 ml of 50% H_2_SO_4_ at room temperature for 2 h, followed by washing with water until pH 6.5–7.0. The suspension was passed through a 0.053 mm mesh sieve (Der Shuenn, Taiwan) to remove large particles. Afterward, the suspension was centrifuged at 6000 rpm for 10 min at 4 °C. The pellet containing colloidal chitin was recovered and stored at 4 °C until use. Glycol chitin (EG-chitin) was prepared using the method (Yamada and Imoto 1981).

### Production and purification of chitinase

*T. virens* strain mango (10^5^ cfu/ml of spores) was cultured in a chitin-containing medium (one liter contained 15 g of colloidal chitin, 0.7 g of K_2_PO_4_, 0.5 g of KH_2_PO_4_, 0.5 g of MgSO_4_·7H_2_O, 18 mg of FeSO_4_·7H_2_O, 1.8 mg of ZnSO_4_·7H_2_O), and incubated at 28 °C with shaking for indicated days. *Trichoderma* filtrate was collected followed by precipitation with 80% ammonium sulfate. After centrifugation, the protein precipitate was dissolved in 10 mM Tris–HCl buffer at pH 7, and dialyzed against the same buffer using cut-off 6–8 kDa dialysis membrane (Spectra/Por®) at 4 °C overnight. Then, the supernatant was applied to a chitin-bead affinity column (Biolabs). After washing out the unbound protein with 10 mM Tris–HCl at pH 7.5, chitinase was eluted with 10% acetic acid buffer. The collected chitinase was dialyzed against 10 mM Tris–HCl at pH 7.5. The activity assay was subsequently performed. Otherwise, it was stored at − 20 °C until use.

### Identification of protein by MALDI/MS

Protein band in SDS-PAGE gel was manually excised and ground into pieces. After washed with 50% acetonitrile and 50% acetonitrile/25 mM ammonium bicarbonate, the protein was in-gel reduced and alkylated in 25 mM ammonium bicarbonate buffer containing 10 mM dithiothreitol and 55 mM iodoacetamide. Then, the protein was digested at 37 °C overnight by 0.1 mg of porcine trypsin (Promega, Madison, WI, USA). The tryptic peptides were subsequently extracted from the gel by 50% acetonitrile/5% formic acid, followed by MALDI/MS analysis using a quadrupole-time-of-flight (Q-TOP) mass spectrometer (Micromass Q-T of Ultima, Manchester, UK) in the proteomics Research Core Laboratory at National Cheng-Kung University, Taiwan.

### Enzyme activity assay

NAGase activity was usually performed by using *p*NP-NAG as the substrate. 10 μl of protein sample was mixed with 50 μl of 50 mM phosphate buffer at pH 5, containing 300 µg/ml *p*NP-NAG. After incubation at 65 °C for 30 min, 50 μl of 0.4 M Na_2_CO_3_ was added to stop the reaction. The absorbance of the mixture was measured at 405 nm to determine the amount of *p*-nitrophenol released according to a standard curve of *p*-nitrophenol. One unit of NAGase activity corresponded to the amount of enzyme required to produce 1 µmol of *p*-nitrophenol min^−1^. For substrate specificity, 1.5% of various substrates including chitin, EG-chitin or CMC were used. After incubation at 40 °C for 24 h, the release reducing sugars were quantified by the DNS method (Ghose 1987).

The fluorometric assays were performed to determine endochitinase activity using a 4-methylumbelliferyl-ß-D-N, N’, N’’-triacetyl chitotriose (Sigma) as subtracts. Following the reaction at 37 °C for 1 h, the released 4-methylumbelliferone (4-MU) was estimated by a spectrofluorometer (Beckman, Fullerton, USA) at an excitation of 360 nm and an emission of 465 nm.

### TLC and HPLC analysis of hydrolytic products

The purified NAGase (50 mU) was incubated in 200 µl of 50 mM phosphate buffer (pH 5) containing 1.5% colloid chitin. Then, the hydrolytic products were analyzed by TLC and HPLC. Using a solvent system, butanol-acetic acid–water (2:1:1, v/v/v), the aliquots of hydrolytic products were spotted onto a TLC silica gel plate (Merck, Damstadt, Germany). The plates were sprayed with solution, containing 1% KOH, 2.5% acetone, 4% ethanol in butanol, followed by heating in an oven at 100 °C for 5 min. Afterward, the plates were sprayed with solution containing 0.4% (w/v) dimethylamino benzaldehyde, 12.5% ethanol, 12.5% HCl and 75% butanol, heating in an oven at 100 °C for 5 min. The hydrolytic products were also subjected to HPLC analysis using a PolySep-GFC-P 2000 column (Phenomenex, USA) with running solution, acetonitrile: water (3:2) at 0.8 ml/min of flow rate under OD_230_ detection using commercial GlcNAc for comparison.

### Antifungal activity assay

To obtain sclerotial bodies, *Sclerotium rolfsii* was cultured on potato dextrose agar for 2–3 weeks. Two pieces of sclerotial bodies from *S. rolfsii* was inoculated into 1 ml potato dextrose broth with or without the purified NAGase. Six pieces of sclerotial bodies were used for each treatment. After incubation at 28 °C for 24–36 h with shaking, the sclerotial bodies were moved to the plate. The hyphal growth inhibition by the purified protein was observed and photographed. The mycelium length was recorded.

### RNA isolation, PCR cloning and RT-PCR analysis

The harvested mycelia of *T. virens* strain mango was frozen with liquid nitrogen, and subsequently ground into a fine powder. For total RNA isolation, 0.1 g of powder sample was mixed with 1 ml of TRIzol reagent (Invitrogen, CA, USA), according to the manufacturer’s instructions. The mixture was stand at room temperature for 5 min, followed by mixing with 200 µl of chloroform. After centrifugation, the aqueous phase was recovered. RNA was precipitated with two volume of ethanol (> 99.8%), rinsed with 70% ethanol and dried on air. Finally, RNA was dissolved in 40 µl of water pretreated with DEPC.

The first strand cDNA was synthesized using SuperScript™ III reverse transcriptase (Invitrogen, CA, USA), and was used as templates for the following PCR cloning of *TvmNAG2* or RT-PCR analysis. Based on DNA sequence of *nag2* from *T. virens* Gv29-8 (*TvNag2*, accession number, XM_014099474), the degenerate primers were designed (forward primer dpNAG2-F, 5’-CTG TGG CCC GTG CCG ANN-3’; reverse primer dpNAG2-R, 5’- TCA GTA ATT CCC TGA CTC ACN-3’). After cloning and sequence analyses, the DNA fragment coding for TvmNAG2 without signal peptide was obtained.

For RT-PCR analysis of *TvmNAG2*, conserved degenerate primer TvNAG-midF, 5- GCG ACC CGA CCA AGA ACT GNN -3’; and reverse primer 5’-TCA GTA ATT CCC TGA CTC ACC G-3’ were used. For RT-PCR analysis of *nag1*, conserved degenerate primer TvNAG-midF; and reverse primer, 5’-TTA GGT GAA CAG CGT GCA AGN-3' were used. Both DNA fragments (~ 350 bp) was separately subcloned into pGEM-T vector, followed by sequencing to confirm they belonged to *TvmNAG2* and *nag1.* The primers for actin, 5’-ATGTGCAAGGCCGGTTC-3’ and 5’-GTCTCGAAGACGATCTGG-3’ were used and the expected PCR product was around 350 bp as well.

### Sequence analysis

The similarity searches were accomplished via BLAST network at NCBI. The alignment of selected sequences was performed with CLUSTAL O (1.2.4) multiple sequence alignment at EMBL-EBI, and then modified.

## Results

### Production, purification and identification of NAGase

**Fig. 1:**
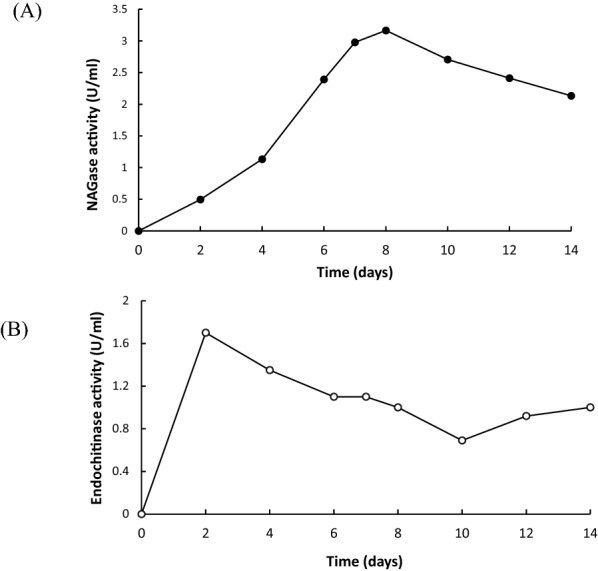
Induced production of endochitinase and NAGase. *T. virens* strain mango was cultured in a colloid chitin-containing liquid medium followed by the determination of **A** NAGase and **B** endochitinase activity

**Fig. 2:**
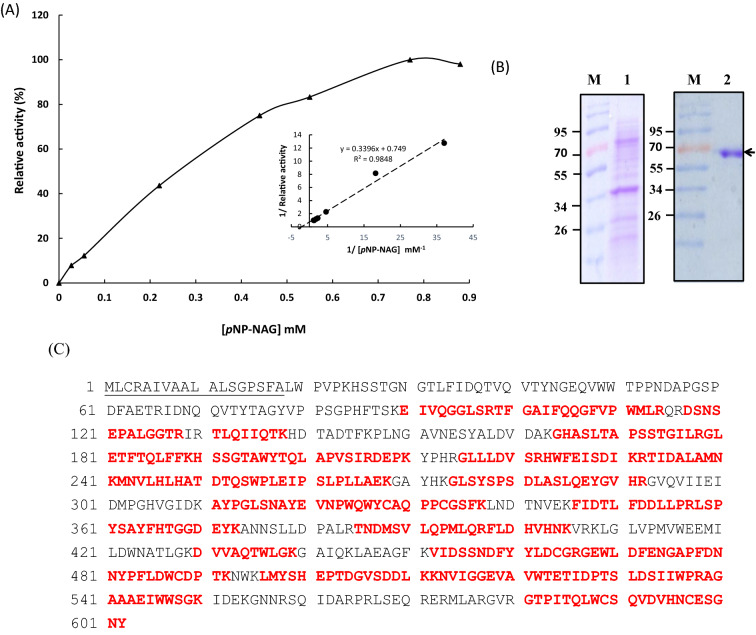
Kinetic parameter and SDS-PAGE analysis of TvmNAG2, and protein sequence of TvNag2. **A** The activity of TvmNAG2 was determined using different concentrations of substrate, *p*NP-NAG. Michaelis–Menten and Lineweaver–Burk graphs were then plotted. **B** SDS-PAGE analysis: lane 1, the crude protein from the precipitation of 8-days *T. virens* filtrate with 80% (NH_4_)_2_SO_4_; lane 2, the affinity-purified TvmNAG2; M, protein marker. **C** TvmNAG2, lane 2 from **B**, was matched to *T. virens* Gv29-8 Nag2 (TvNag2, accession number, XM_014099474) after MALDI/MS analysis. The matched peptides of TvmNAG2 were marked in red

A *Trichoderma* isolate*, T. virens* strain mango with high chitinase activity on a plate-based survey was cultured in a liquid medium containing colloid chitin. The maximum endochitinase activity was detected after cultivation for 2 days; while the NAGase activity was reached to maximum after cultivation for 8 days (Fig. 1). The filtrate of *T. virens* strain mango cultured for 8 days was collected, followed by purification of chitinase. The crude proteins were precipitated with 80% ammonium sulfate. After centrifugation and dialysis, the crude proteins were directly purified by chitin-bead affinity chromatography. The yield and purification folds of *T. virens* strain mango NAGase was summarized in Table 1. *T. virens* NAGase activity was detected during the purification, while no endochitinase activity was monitored after chitin-bead affinity purification. *T. virens* NAGase was purified to 38.8 folds with ~ 2.64% recovery. The specific activity was 10,698.3 U/mg using *p*NP-NAG as the substrate. After Lineweaver–Burk graph was plotted, *K*m was determined to be ~ 0.45 mM (Fig. 2A).

The native NAGase was purified to homogeneity with molecular mass of ~ 68 kDa established on SDS-PAGE analysis (Fig. 2B). The protein band was subjected to protein identification analysis. MALDI/MS analysis indicated that it was corresponded to the predicted Nag2 from *Trichoderma* spp., particularly matched to Nag2 from *T. virens* Gv29-8 (TvNag2, accession number: XM_014099474) with 61% of protein sequence coverage (Fig. 2C). Accordingly, the purified native NAGase of *T. virens* strain mango was identified as a Nag2, termed TvmNAG2.

### Effect of pH and temperature on activity

**Fig. 3:**
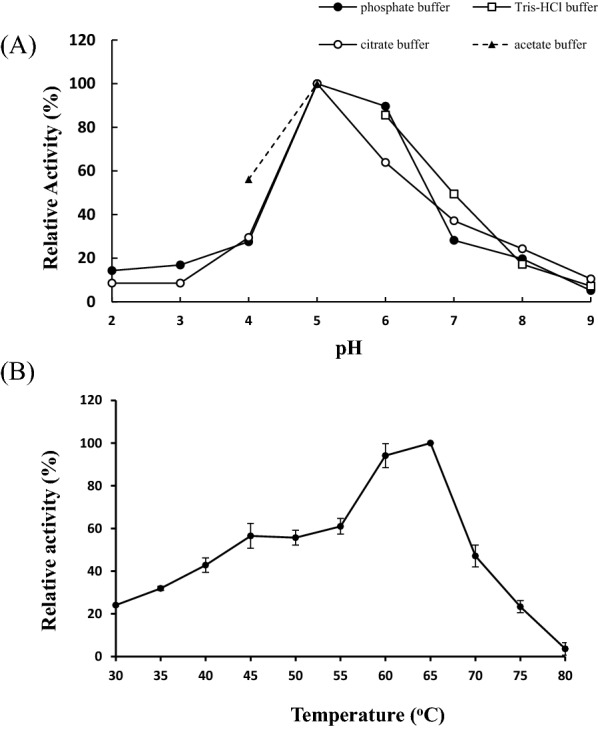
Optimal pH and temperature of TvmNAG2. **A** To determine the optimal pH of TvmNAG2, NAGase activity assay was performed at various pH of buffer (final 41.7 mM) including citrate buffer, acetate buffer, Tris buffer or phosphate buffer at 65 °C for 30 min. **B** To determine the optimal temperature of TvmNAG2, NAGase activity assay was performed at different temperature, 30–80 °C for 30 min at pH 5

The optimal pH and temperature for activity assay of TvmNAG2 was examined using *p*NP-NAG as the substrate. For the determination of optimal pH, four buffers were used, including citrate (buffer range, pH 3–6), phosphate (buffer range, pH 5–8), acetate (buffer range, pH 3.6–5.6) and Tris-HCl (buffer range, pH 7–9). The activity was assay under different pH and buffer system, even not within their pH buffer range. To be noted, the optimal pH 5 was almost the same, as shown in Fig. 3A. The activity decreased more sharply using phosphate buffer at pH 4 and 7, compared with acetate buffer (pH 4) and Tris buffer (pH 7). The purified TvmNAG2 had the highest activity at pH 5 (Fig. 3A). More than 85% of NAGase activity was detected at pH 6, while less than 30% of activity was monitored as at pH < 4 or pH ≥ 8 (citrate buffer at pH 6 and acetate buffer at pH 4 was the exception).

Figure 3B showed that the optimal assay temperature of TvmNAG2 was 60–65 °C. And ~ 60% of NAGase activity was detected as the assay temperature was 45–55 °C. The activity was dramatically decreased as the assay temperature was higher than 65 °C.

**Fig. 4:**
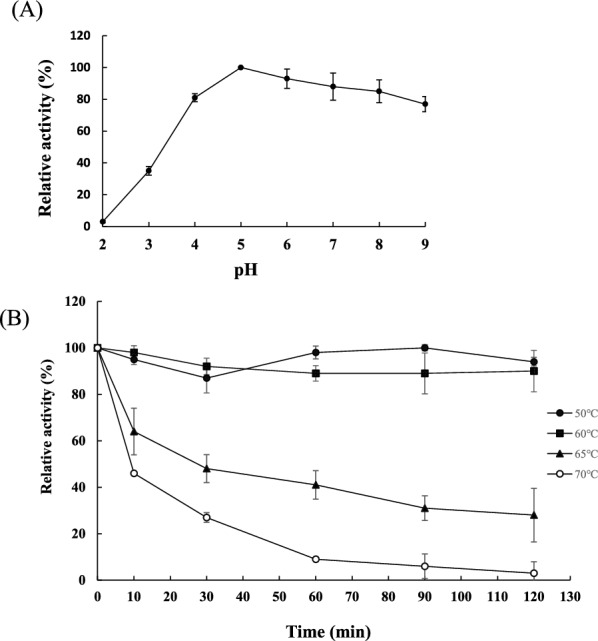
pH and thermal stability of TvmNAG2. **A** For pH stability, TvmNAG2 was in 10 mM phosphate buffer at various pH values for 1 h, followed by activity assay. **B** For thermal stability, TvmNAG2 was incubated at various temperatures (50–70 °C) for 0–120 min, followed by activity assay. The activity assay was performed at 65 °C and pH 5

To examine pH effect on TvmNAG2, it was incubated at diverse pH condition for one hour, followed by determination of NAGase activity at pH 5 and 65 °C. TvmNAG2 was very stable at pH 5.0, and more than 80% of activity was retained between pH 4 and 9. The activity decreased dramatically as pH was lower than 3.0 (Fig. 4A). The thermal stability of TvmNAG2 was evaluated. TvmNAG2 was treated at different temperatures, 50–70 °C for 0–120 min, followed by activity assay. TvmNAG2 exhibited thermal stability and retained more than 90% activity after treatment at 60 °C for 120 min (Fig. 4B). The protein lost its activity to less than 30% after incubation at 70 °C for 30 min.

### Substrate specificity of TvmNAG2, and its hydrolytic product using colloidal chitin

The colloidal chitin, powdery chitin, glycol chitin, chitosan (85% of deacetylation), CMC, starch at concentration of 1.5% each were provided as the substrate. TvmNAG2 exhibited the highest activity toward EG-chitin (relative activity, 100%), followed by colloidal chitin (47.6%). The other polysaccharides could not be hydrolyzed by TvmNAG2.

**Fig. 5:**
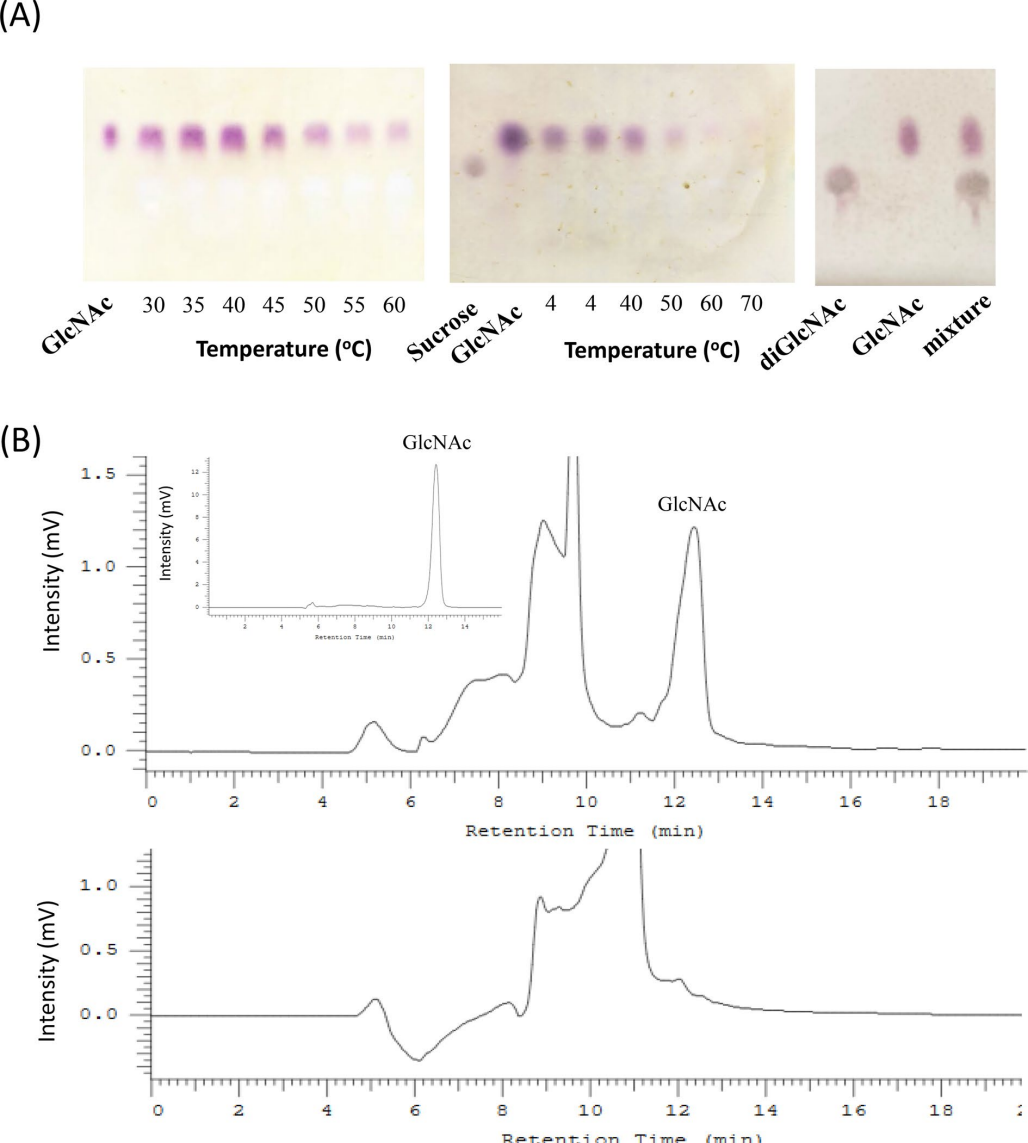
TLC and HPLC analyses of product hydrolyzed by TvmNAG2 using colloidal chitin as the substrate. **A** TvmNAG2 was incubated with 1.5% colloidal chitin at 30–60 °C for 20 h, followed by TLC analysis (left). TvmNAG2 was treated at 4, 40, 50, 60 and 70 °C for 2 h. Afterward, TvmNAG2 was incubated with 1.5% colloidal chitin at 40 °C for 20 h, followed by TLC analysis (middle). diGlcNAc (*N*, *N*’-diacetylchitibiose), GlcNAc and mixture (diGlcNAc and GlcNAc) was on TLC analysis as well (right). **B** TvmNAG2 was incubated with 1.5% colloid chitin at 37 °C for 20 h, followed by HPLC analyses (upper). For comparison, GlcNAc was used as the standard sample. The colloid chitin was as the control (below)

TLC and HPLC analyses were performed to evaluate the end product using the colloidal chitin as substrate. The result with TLC analyses suggested that TvmNAG2 hydrolyzed the substrate to produce GlcNAc (Fig. 5A). The optimal temperature to yield the catalytic product of the colloidal chitin by TvmNAG2 was at 40 °C, when the catalytic reaction last for 20 h (Fig. 5A). It seemed that TvmNAG2 lost its ability to hydrolyze colloidal chitin completely after treatment at 60 °C for 120 min. Moreover, the presumed GlcNAc peak appeared in HPLC analyses with the shoulder on the left side (Fig. 5B), which probably was from the background of colloidal chitin.

### Effect of ions, surfactants and EDTA on NAGase activity

**Fig. 6:**
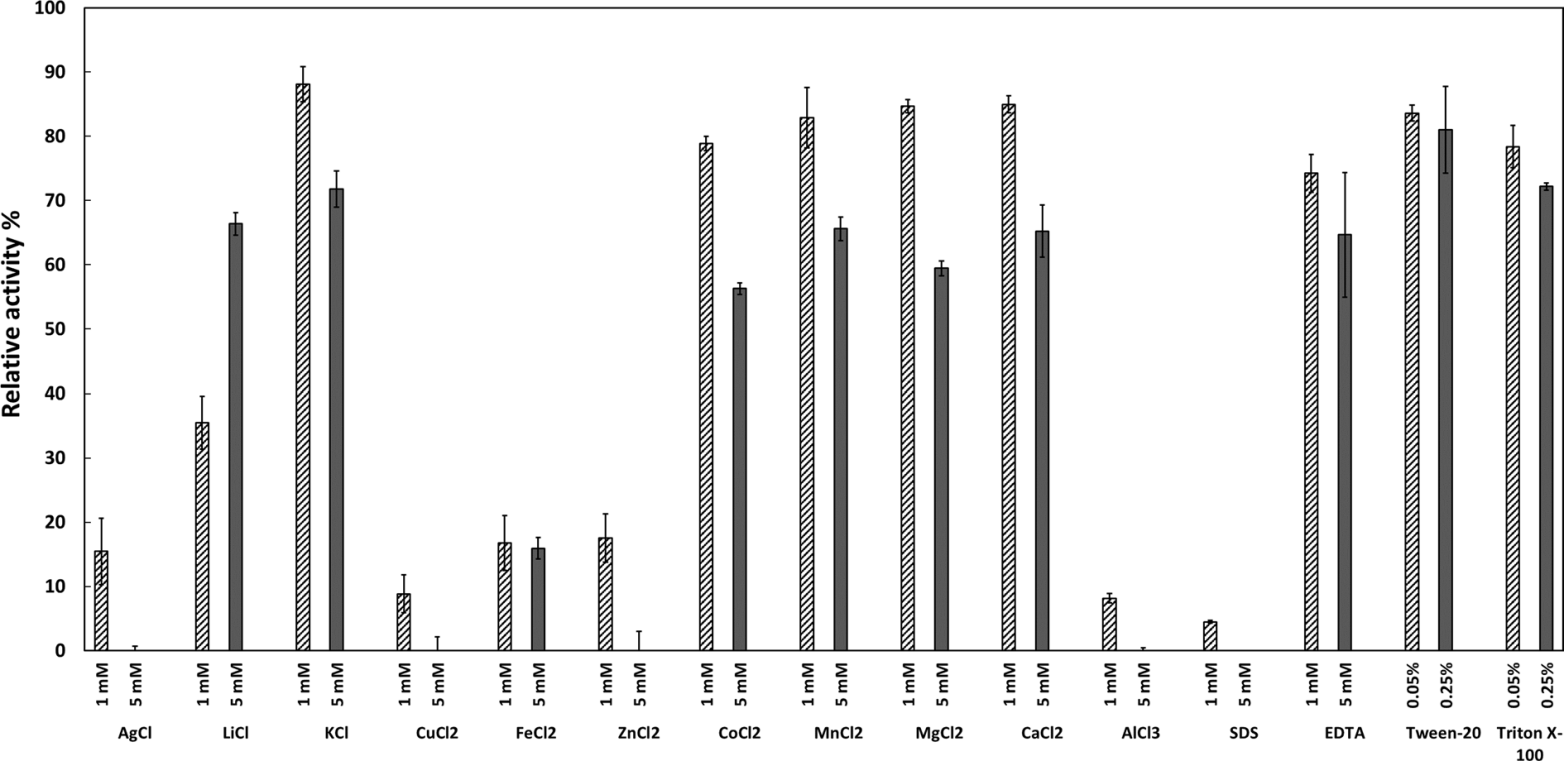
Effect of ions, surfactants and EDTA on NAGase activity. TvmNAG2 was incubated with additional metal ions, surfactants or EDTA at the indicated concentration, followed by activity assay. The activity of TvmNAG2 without any effector was as 100% activity. The experiments were repeated triple with standard deviation

The activity of TvmNAG2 was affected by the examined metal ions, surfactants and EDTA (Fig. 6). Ag^+^, Fe^2+^, Cu^2+^, Zn^2+^, Al^3+^ or SDS have strong inhibitory effect on the activity, of which < 20% remained at a concentration of 1 mM for each. 5 mM of Li^+^ reduced the activity to less than 40%. The activity was declined to 74% and 64% by EDTA at 1 and 5 mM of concentration, respectively. ~ 80% activity remained with Tween-20 or Triton X-100 at a concentration of 0.05%. Moreover, citrate (pH 5) stimulated the activity to ~ 2.4 folds at 41.7 mM of final concentration, compared to phosphate buffer and acetate buffer (pH 5) (data not shown). Citrate buffer could not enhance the activity at pH ≤ 4.

### Protease resistance

**Fig. 7:**
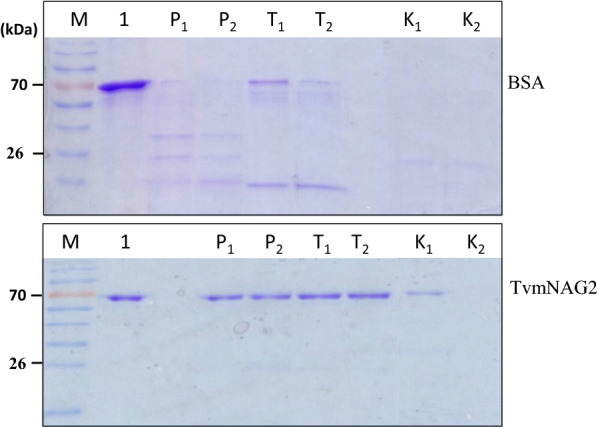
Effect of protease on TvmNAG2. 2 µg of either TvmNAG2 (below) or BSA (upper) was treated with commercial protease, including papain, trypsin, or protease K at 25 °C for 1 h, followed by SDS-PAGE analyses. Lane 1, without protease treatment. Lane P_1_ and P_2_, papain treatment; lane T_1_ and T_2_, trypsin treatment; lane K_1_ and K_2_, protease K; whereas _1_, _2_ represented 0.6 and 1 µg of commercial protease added into the reaction, respectively

TvmNAG2 was treated with protease, papain, trypsin or protease K at 25 °C for 1 h. Papain, trypsin and protease K belong to the member of cysteine proteases, serine protease, and serine protease, respectively. TvmNAG2 showed protease resistance to papain, trypsin and protease K, compared with BSA (Fig. 7). TvmNAG2 remained the intact on SDS-PAGE with 100% NAGase activity (data not shown), after digestion with papain or trypsin. It was more resistant to the digestion by papain and trypsin than protease K.

### Inhibitory effect on the mycelium growth of S. rolfsii

**Fig. 8:**
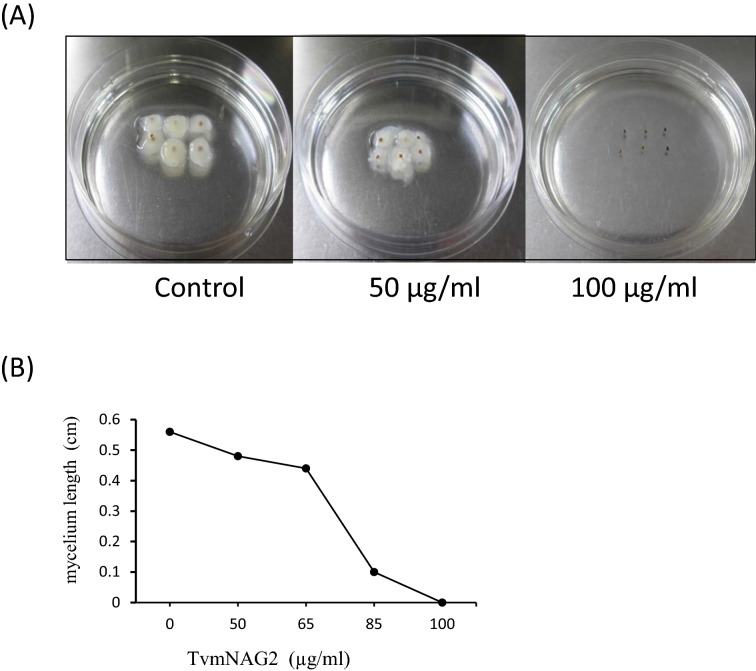
Growth inhibition of *S. rolfsii* mycelium by TvmNAG2*.* **A** The sclerotial bodies were grown in a medium containing TvmNAG2 at 50 or 100 µg/ml overnight. Then, the sclerotial bodies was placed on the plates, and recorded. Control, without any TvmNAG2. **B** The effect of TvmNAG2 at 0–100 µg/ml on the mycelium length of *S. rolfsii* was studied

*S. rolfsii* found in the warm temperate regions could cause southern blight damage to the legumes, crucifers and cucurbits seriously. The purified native TvmNAG2 could retard the growth of *S. rolfsii* mycelium, as shown in Fig. 8A. 100 μg/ml of TvmNAG2 could completely inhibit the mycelium growth (Fig. 8).

### Enzyme stability during storage

**Fig. 9:**
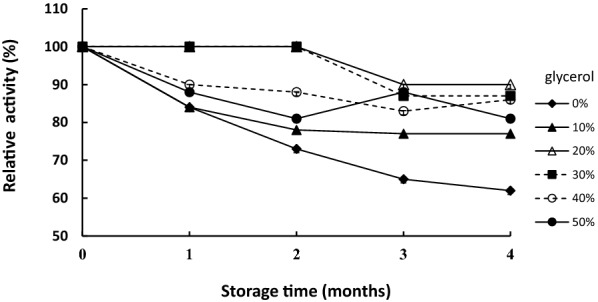
The effect of glycerol on TvmNAG2 during the storage at − 20 °C. TvmNAG2 containing 0–50% glycerol was stored at − 20 °C for 1–4 months, followed by activity assay

Glycerol may have positive impact on the enzyme stability stored at − 20 °C, like most restriction enzymes commercially available. As shown in Fig. 9, 0–50% glycerol was examined its effect on TvmNAG2 stored at − 20 °C for 1–4 months. Without any glycerol, TvmNAG2 lost 30 ~ 40% activity after storage at − 20 °C for 2–4 months. And 20–30% glycerol could preserve the enzyme to have > 85% of protease activity within 4 months at − 20 °C. Moreover, TvmNAG2 first via sterile filter with 0.2 μm membrane was stored at − 20, 4 and 25 °C for 2 months. ~ 20 and ~ 30% activity was lost as it was stored at 4 and − 20 °C, respectively. TvmNAG2 was stable for 2 months at 25 °C under the sterile condition, ~ 90% activity of TvmNAG2 remained (data not shown).

### Cloning and expression of TvmNAG2

**Fig. 10:**
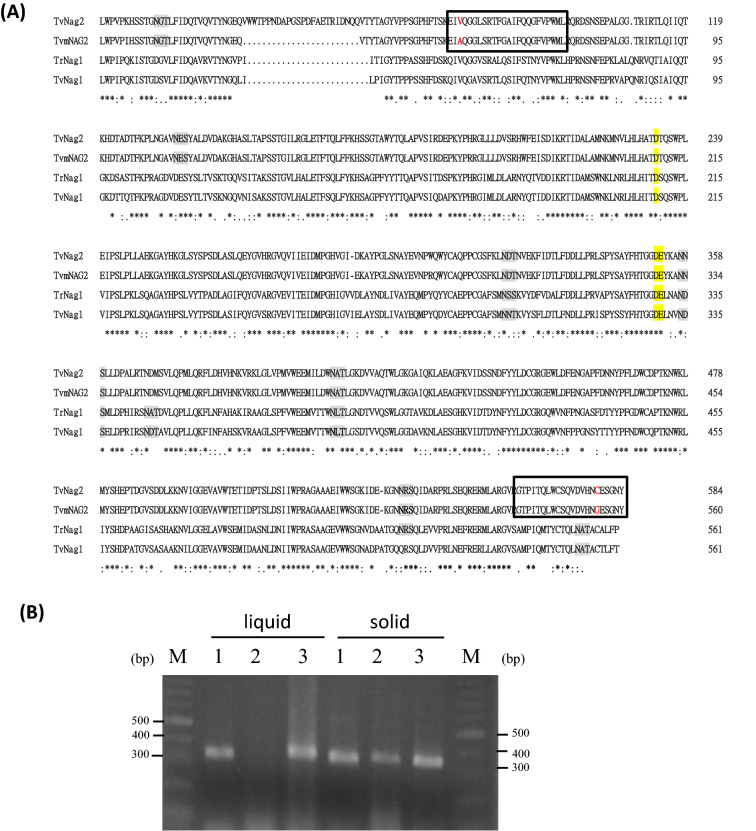
Deduced protein sequence alignment of *nag1 and nag2*, and expression of *nag1* and *TvmNAG2*. **A** The deduced TvmNAG2, TvNag1, TvNag2 and TrNag sequences from *T. virens* strain mango, *T. virens* strain Gv29-8, *T. virens* strain Gv29-8 and *T. reesei* strain QM6a were aligned (accession number of OL456168, XM_014095216, XM_014099474, and XM_006963001, respectively). The conserved E (Glu) and D (Asp) in active site were highlight. Two amino acids of deduced TvmNAG2 marked in red were different from the amino acids within the matched TvNag2 peptides of MALDI/MS analyses. Such peptides were boxed. Putative glycosylated sites, NxS/T were shaded. **B** The expression of *TvmNAG2* and *nag1* from *T. virens* strain mango in liquid and solid cultures were analyzed by RT-PCR. Line 1 and 4, *actin*; line 2 and 5, *nag1*; line 3 and 6. *TvmNAG2*

According to the sequence of *nag2* from *T. virens* Gv29-8 (*TvNag2*), the primers containing 3’ terminal degenerate nucleotide were designed. A DNA fragment, coding for *T. virens* strain mango Nag2 (presumably TvmNAG2) without signal peptide, was successfully obtained by PCR. The encoded TvmNAG2 shares 94.7% identity with the deduced protein sequence of *TvNag2*, as shown in Fig. 10A. MALDI/MS data of native TvmNAG2, matched to TvNag2, was found in deduced protein sequence of *TvmNAG2* (Fig. 2B), except for two sites, A50 and G555 of encoded TvmNAG2 (Fig. 10A). The encoded TvmNAG2 was aligned with Nag1 and Nag2 from *T. virens* Gv29-8 (TvNag1 and TvNag2), as well as Nag1 from *T. reesi* (TrNag1), of which recombinant protein was characterized (Chen et al. 2015). TvmNAG2 shares 57.2% identity with TrNag1, and 57.7% identity with TvNag1. The encoded TvmNAG2 comprised D209, D328 and E329, which are important for catalytic activity of NAGase (Lemieux et al. 2006; Vocadlo and Withers 2005). NAGase of GH20 family employ retaining mechanism of catalysis, and the conserved Glu and Asp were found in all aligned sequences (Fig. 10A). After cloned into pET21b expression vector, the recombinant TvmNAG2 was overexpressed in the inclusion bodies of *E. coli* BL21(DE3) after induction with 1 mM IPTG at 37 °C for 4 h (data not shown). The expression of the recombinant in the supernatant of *E. coli* was failed under the induction conditions at 15 °C.

The expression of *nag2* (*TvmNAG2*) and *nag1* from *T. virens* strain mango were analyzed by RT-PCR using the primers designed according to the conserved region and 3’ terminal of open reading frame. Only *TvmNAG2* was expressed in *T. virens* liquid cultures containing colloidal chitin, but not *nag1*, as shown in Fig. 10B. Both of *nag1* and *TvmNAG2* were expressed, when *T. virens* was cultured on plates containing colloidal chitin.

## Discussion

Polymerzation of GlcNAc by 1,4-ß-linkages leads to form chitin, which is mainly catalyzed by chitin synthases and degraded by chitinolytic enzymes like endochitinase, chitobiosidases or NAGase. And among them, NAGase has been revealed to play important roles for its functions, such as hydrolysis of GlcNAc-containing oligosaccharides and proteins to yield GlcNAc (Intra et al. 2008; Slámová et al. 2010; Zhang et al. 2018).

According to the genome-wide analyses, two putative genes, *nag1* and *nag2* of GH20 family encoding NAGase*,* are in the genome of *T. virens*, *T. atroviride* or *T. reesei* (Kubicek et al. 2011). *Trichoderna* NAGases in the native or recombinant forms have been reported and summarized in Table 2. The reported NAGase has a molecular mass between 28 and 93 kDa. The protein identity of most native *Trichoderma* NAGases reported previously was not known yet. Moreover, compared to Nag1, little was known with the catalytic properties of Nag2.

*T. reesei nag1* was cloned and homologous overexpressed in *T. reesei strain* RutC30ΔU3 with the strong cellobiohydrolase promoter (Chen et al. 2015). The recombinant *T. reesei* rNag1 displayed optimal pH value of 4.0, and optimal temperature at 60 °C for the reaction using *p*NP-NAG as substrate. It showed > 60% activity at pH 3.5 ~ 6. *T. reesei* rNag1 was examined for its stability at pH 3–9. At least 80% activity was detected as *T. reesei* rNag1 was treated at pH 4–6 for 1 h. Its activity declined a lot under pH > 6.0, and less than 20% activity was remained after treatment at pH 9. The recombinant exhibited thermal stability, which remained ~ 80% and ~ 60% activity after treatment at ~ 60 °C for 2 h and 8 h, respectively.

In this study, a native TvmNAG2 from *T. virens* strain mango was purified and was matched to Nag2 from *T. virens* Gv29-8 with 61% of protein sequence coverage. Using *p*NP-NAG as substrate, the optimal pH of TvmNAG2 for activity assay was pH 5, and its optimal temperature was 60–65 °C, which assay duration was 30 min. To be noted, the optimal temperature of TvmNAG2 to hydrolyze chitin for 20 h was 40 °C, according to amount of expected GlcNAc on TLC analyses. After treatment at 60–70 °C for 2 h, the ability of TvmNAG2 to hydrolyze chitin was almost lost. The optimal temperature was usually as a parameter in enzyme property. Recently, it was reported that optimal temperature is a relative term related to the duration and enzyme concentration in assay/catalytical reaction (Almeida and Marana 2019). It was also shown that some enzyme exhibited substrate-dependent optimal temperature, such as peroxidase from *Bacillus subtilis* (Min et al. 2015).

Regarding to pH stability of TvmNAG2, > 80% of activity was remained after it was treated at pH 4–9 for 1 h. Its activity decreased dramatically under pH < 4.0 for 1 h. TvmNAG2 showed broader pH stability than *T. reesei* rNag1, pH 4–6. TvmNAG2 retained more than 90% activity after treatment at 60 °C for 2 h. The protein lost its activity to less than 30% after incubation at 70 °C for 30 min. TvmNAG2 was demonstrated to be thermostable, better than *T. harzianum* (strain 39.1) NAGase, *T. reesei* rNag1 and others in Table 2.

*T. virens* strain mango *nag2* gene coding for Nag2 (TvmNAG2, presumably) was obtained by PCR. The deduced protein sequences of *nag2* from *T. virens* strain mango and strain Gv29-8 share 95% identity to each other. The molecular mass of native TvmNAG2 was established to be ~ 68 kDa on SDS-PAGE. The predicted mature TvmNAG2 has molecular mass of 62.7 kDa, smaller than its native form. Five NXS/T of putative N-glycosylation sites are found in the deduced protein sequence of *TvmNAG2*. Whether TvmNAG2 is a glycoprotein remains further study. *T. harzianum* strain 39.1 NAGase was demonstrated to be a glycoprotein by using tunicamycin, an inhibitor of protein *N*-glycosylation (Ulhoa et al. 2001). Using gel filtration, the molecular mass of underglycosylated and glycosylated forms of *T. harzianum* NAGase was 110 and 124 kDa, respectively. The glycosylated form was more thermostable and trypsin-resistant than underglycosylated form.

TvmNAG2 was resistant to the proteolysis by papain or trypsin. *T. harzianum* strain 39.1 NAGase also showed trypsin-resistance (Ulhoa et al. 2001). The effect of the metal ions and some chemical reagent on the activity of TvmNAG2 was examined. SDS and certain metal ions significantly impeded its activity, remained much less than 20% activity, Al^3+^(8.2% activity remain), Cu^2+^(8.9%), and Ag^+^ (15.5%) at a concentration of 1 mM. Overall, the metal ions had less effect on *T. reesei* rNag1, for example, Al^3+^(68% activity remain), Cu^2+^ (80%), Zn^2+^ (86%) at 1 mM (Chen et al. 2015).

NAGases are widely distributed in most organisms, except for the domain of archaea. The physiological and functional roles of NAGases are diverse, related to the organisms and the cellular location (Slámová et al. 2010). The deduce proteins of the full length *nag1*and *nag2* from *Trichoderma* spp. contain signal peptide, suggesting their extracellular location. The reported *Trichoderma* NAGases, including TvmNAG2 in this study were found in the culture filtrate. RT-PCR analysis showed that *TvmNAG2* was expressed in *T. virens* strain mango cultured on the plate or in chitin-containing liquid medium; while *nag1* was only expressed in *T. virens* strain mango grown on the chitin-containing plate. Upon different cultivation, how the *nag1* and *TvmNag2* at transcript and protein level were regulated in *T. virens* strain mango is remained further study. It was reported that the water content of the solid-state culture caused differently expression of glucoamylase-encoding gene in *Aspergillus oryzae* (Kobayashi et al. 2007). The cultivation methods of microorganisms such as *Clostridium perfringens* affect their gene expression profile through diverse regulation of transcription (Soncini et al. 2020).

*TvNag1* transcript was largely abundant in *T. virens* 29–8 grown in a liquid medium containing 0.5% chitin; whereas *TvNag2* transcript was not detected, based on Northern blot analyses (Kim et al. 2002). And 1.0% fungal cell wall could induce much more the expression of *TvNag1* than *TvNag2*. Two NAGases, EXC1Y and EXC2Y were purified, and the corresponding genes and promotors were studied from *T. asperellum* (Ramot et al. 2004). EXC1Y and EXC2Y, active as homodimer, are the member of Nag1 and Nag2, respectively. However, the enzymatic properties of both EXC1Y and EXC2Y were not further characterized. A knockout mutant of *exc2y* was studied, suggesting that *exc2y* is not essential for the growth and biocontrol function of *T. asperellum* (Ramot et al. 2004). Using the knock-out study with Δnag1 and Δnag2, NAGase (either Nag1 or Nag2) were demonstrated to be are necessary for the growth of *T. atroviride* on chitin or chitobiose (López‐Mondéjar et al. 2009).

Extracellular NAGase from *Trichoderma* spp. may play a defense role to against other chitin-containing microorganisms including phytopathogenic fungi. *T. atroviride* Nag1 was demonstrated to be essential for chitinase induction by chitin, and the disruption-*nag1* reduced 30% ability of biocontrol *T. atroviride* against infection by *Rhizoctonia solani* and *Sclerotinia sclerotiorum* (Brunner et al. 2003). The physiological role of TvmNAG2 was still unclear. TvmNAG2 was demonstrated to have antifungal activity, inhibiting the hyphal growth of *S. rolfsii*.

More study was focus on Nag1 than Nag2 from *Trichoderma* spp., perhaps due to the significant induction of *nag1* under the examined conditions (Kim et al. 2002; Ramot et al. 2004). To our best knowledge, it is the first study to characterize the catalytic activity of Nag2 under various conditions. And herein, the results presented that TvmNAG2 has promising potential for further application, due to its thermal and pH stability, protease resistance, anti-fungal activity and perhaps GlcNAc production.

**Table 1:** Purification of NAGase from *T. virens*. *T. virens* strain mango were cultured in a liquid medium containing chitin for 8 days, followed by the purification steps, including ammonium sulfate precipitation, and a chitin-bead affinity chromatography, followed by NAGase activity assay using *p*NP-NAG as substrate

Procedure	Total volume (ml)	Total activity (U)	Total protein (mg)	Specific activity (U/mg)	Purification (folds)	Yield (%)
Culture filtrate	450.0	2,430.60	8.820	275.6	1.0	100.00
(NH_4_)_2_SO_4_ precipitation	1.9	3,772.80	2.420	1,558.6	5.7	155.00
Chitin-bead chromatography	60.0	64.19	0.006	10,698.3	38.8	2.64

**Table 2:** Properties of the reported NAGase from various *Trichoderma* spp.

*Trichoderma* spp.	Name of enzyme; form; expression level	Mw (kDa)	Optimal pH; optimal temperature	pH tolerance; temperature tolerance (% activity remained)	Specify activity^b^ (U mg^−1^ min^−1^); purification folds	*K*m^b^	Refs.
*T. virens* strain mango	TvmNAG2; native; 5.4 U/ml	~ 68	pH 5 (pH 5–6, > 90% activity); 60–65 °C	pH 4–9 at 25 °C for 60 min (≧80%); 60 °C at pH 7 for 120 min (~ 85%)	10,698.3; 38.8	0.45 mM	In this study
*T. reesei* RutC30ΔU3	Nag1; Recombinant; 499.85 U/ml	~ 80	pH 4 (pH 3.5–6, > 60% activity); 60 °C	pH 4–6 for 60 min (≧80%); 60 °C for 120 min (~ 80%), 60 °C for 8 h (~ 60%)	319.89	69.41 ± 4 M	(Chen et al. 2015)
*T. harzianum* 1051/ NAGase	NAGase	36	pH 4; 37 °C	nd; 50 °C at pH 4 for 60 min ~ 50%	nd	8.06 mM	(De Marco et al. 2004)
*T. harzianum* Strain 39.1	NAGase; native	64, 118^a^	pH 5.6; 50 °C	nd	nd	1.27 µmol	(Ulhoa et al. 2001)
*T. harzianum* T198	Exochitinase; native	27.5 ~ 28	pH 3.5; 50 °C	pH 2–8 at 37 °C for 60 min (≧50%); 60 °C at pH 5.5 for 60 min (33%)	nd	850 µM ^c^	(Deane et al. 1998)
*T. harzianum* P1	Nag1; native	72	nd	nd	11.8^bd^; 9	nd	(Lorito et al. 1994)
*T. harzianum* AF6-T8	NAGase; native; 3.1 U/ml	~ 69, 150^a^	pH 4–5.5; 50 °C	pH 3–6 at 37 °C for 60 min (≧80%); 65 °C at pH 5.2 for 30 min (~ 50%)	102; 13.2	0.24 mM	(Koga et al. 1991)

*nd* no data

^a^Established by gel filtration

^b^*p*NP-NAG as substrate

^c^Chitotriose as substrate

^d^Specific activity = nkatal mg^−1^; one nkatal corresponds to the release of 1 nmol of *p*-nitrophenol per second

## Abbreviations

NAGase: *N*-Acetylglucosaminidase
GlcNAc: *N*-Acetylglucosamine
GlcN: Glucosamine
*p*NP-NAG: *p*-Nitrophenyl-*N*-acetyl-*β*-D-glucosaminide
CMC: Carboxymethylcellulose
DNS: 3,5-Dinitrosalicylic acid
GH: Glycoside hydrolase
4-MU: 4-Methylumbelliferone
